# Method of Detection of Well-Differentiated Thyroid Cancers in Obese and Non-Obese Patients

**DOI:** 10.1371/journal.pone.0152768

**Published:** 2016-04-04

**Authors:** Jonathan Zagzag, Michael K. Malone, Melissa A. Lopresti, Jennifer B. Ogilvie, Kepal N. Patel, Keith S. Heller

**Affiliations:** Department of Surgery, Division of Endocrine Surgery, NYU Langone Medical Center, New York, New York, United States; IPATIMUP/Faculty of Medicine of the University of Porto, PORTUGAL

## Abstract

**Background:**

The incidence of well-differentiated thyroid cancer (WDTC) is increasing rapidly. Many authors feel that this increase is due to over-diagnosis and that one of the contributing factors is the increasing use of various imaging studies. The rate of obesity has also been increasing in the United States. It has been suggested that patients with an increased body mass index (BMI kg/m^2^) have a higher incidence of WDTC than patients with normal BMI. One might hypothesize that thyroid nodules are more difficult to palpate in obese patients and that as more cancers are detected by imaging the apparent rate of increase in WDTC in obese patients would appear to be greater than in non-obese patients. This study was undertaken to evaluate this hypothesis by determining if there is any difference in the way thyroid cancers are initially detected in obese and non-obese patients.

**Methods:**

The medical records of all 519 patients with a postoperative diagnosis of WDTC who underwent thyroidectomy at NYU Langone Medical Center from January 1, 2007 through August 31, 2010 by the three members of NYU Endocrine Surgery Associates were reviewed. Patients were divided into Non-obese (BMI<30 kg/m^2^) and Obese (BMI≥30 kg/m^2^) groups. Patients were also divided by the initial method of detection of their tumor into Palpation, Imaging, and Incidental groups.

**Results:**

The final study group contained 270 patients, 181(67%) of whom were in the Non-obese Group and 89(33%) were in the Obese Group. In the Non-obese group, 81(45%) of tumors were found by palpation, 72(40%) were found by imaging, and 28(16%) were found incidentally. In the Obese group, 40(45%) were found by palpation, 38(43%) were found by imaging, and 11(12%) were found incidentally. These differences were not statistically significant (p-value 0.769).

**Conclusion:**

We show that BMI does not play a role in the method of initial detection in patients with WDTC. This suggests that the prevalence of WDTC detected by imaging is not an artifact caused by an increasingly obese population and that any association of WDTC and obesity is not related to the way in which these tumors are detected.

## Introduction

The incidence of well-differentiated thyroid cancer (WDTC) is increasing at a rapid rate, and has nearly tripled in the United States since 1975 [1–5]. Although some authors maintain that a part of this increase is due to an increase in disease burden [2, 4, 6], others feel that most of this increase is a result of over diagnosis [1, 7–12]. It has been suggested that one of the contributing factors to over-diagnosis is the increasing use of various imaging studies [7]. We have previously shown that 46% of WDTCs are detected by imaging, and that this is not limited to small cancers [13].

The rate of obesity has also been steadily increasing in the United States [14]. It has been suggested that patients with an increased body mass index (BMI kg/m^2^) have a higher incidence of WDTC than patients with normal BMI [15–17]. One might hypothesize that thyroid nodules are more difficult to palpate in obese patients and that as more cancers are detected by imaging the apparent rate of increase in WDTC in obese patients would appear to be greater than in non-obese patients. This study was undertaken to evaluate this hypothesis by determining if there is any difference in the way thyroid cancers are initially detected in obese and non-obese patients.

## Materials and Methods

The medical records of all 519 patients who underwent thyroid surgery at NYU Langone Medical Center from January 1, 2007 through August 31, 2010 by the three members of NYU Endocrine Surgery Associates and who had a postoperative diagnosis of WDTC were reviewed. Forty-six patients were excluded because the method of initial detection of the tumor could not be determined. Of the remaining 473 patients, BMI was available in 270.

Height and weight were obtained from the anesthesia pre-operative records. BMI was calculated by the World Health Organization 2004 classification [18]. The 270 patients in whom the BMI was known were divided into 2 groups: those with BMI ≥30 kg/m^2^ (Obese Group) and those with BMI <30 kg/m^2^ (Non-obese Group). A secondary analysis was performed with patients divided among 4 groups: those with BMI <30 kg/m^2^, those with BMI ≥30 and <35 kg/m^2^, those with BMI ≥35 and <40 kg/m^2^, and those with BMI ≥40 kg/m^2^.

Patients were also classified based on the method of initial cancer detection. This data was self reported by the patients, and patients were asked how their tumor was initially detected. If the answer was imaging, then further clarification was obtained to ensure that the imaging study was not ordered to evaluate a previously palpated nodule. Any nodule with appropriate sonograms findings for further workup raised clinical suspicion [19]. The Imaging Group included patients in whom the indication for surgery was a suspicious or malignant cytological finding on fine needle aspiration biopsy of a nodule that was initially detected on an imaging study. The Palpation Group included those patients in whom the indication for surgery was a suspicious or malignant cytological finding on fine needle aspiration biopsy and in whom the patient stated that further diagnostic studies were initiated because a physician had noted an abnormality on physical examination or because the patient or another non-professional had noted a mass in the neck. The Incidental Group included the remaining patients in whom incidental cancers were found on pathological study of the surgical specimen that were not related to the indication for thyroidectomy. In the Incidental Group, indications for surgery included nodules with suspicious cytology that proved to be benign, symptomatic or enlarging nodules with benign cytology, multinodular goiters and thyrotoxicosis.

Tumor size was measured by the pathologist from the unfixed, fresh surgical specimen. In patients with multifocal cancer, the size of the largest tumor was reported.

Statistical analysis was performed using SPSS version 20 for Windows (SPSS, Chicago, IL). Contingency tables were analyzed by chi-squared test and fisher’s exact test and comparison of means was performed with independent samples t-test and one-way analysis of variance (ANOVA). Adjusting for confounders was performed using multinomial logistic regression. Confounders were chosen a-priori and included age, sex, and tumor size. P-values were two sided and values ≤ 0.05 were considered significant.

This study was approved by the NYU Cancer Institute Protocol Review and Monitoring Committee and by the NYU Institutional Review Board. Written informed consent was obtained from all patients involved in the study.

## Results

The characteristics of the 270 patients in whom the BMI was available and the 203 patients in whom it was not are summarized in Table 1. Patients in whom BMI was known were older than those in whom BMI was not known. No other significant differences between these groups were found. Of the cases included in the study 254 (94%) had papillary cancers, 3 (1%) had follicular cancers, 7 (3%) had Hurthle cell cancers, and 5 (2%) had tall cell variants of papillary cancer.

**Table 1:** Patient and Tumor Characteristics.

	BMI Known	BMI not Known	
	270 (57%)	203(43%)	P-value
Average Age	52(±14)	45.5(15.2)	<.01
Female	190(70%)	155(76%)	0.15
Detection Method			
Palpation	121(45%)	97(48%)	
Imaging	110(41%)	74(37%)	
Incidental	39(14%)	32(16%)	0.64
Average Size (mm)	17 (±13)	16 (±14)	0.65
Tumors <1cm	83(31%)	73(36%)	0.23

n = 473.

The patients who had their tumors found by imaging all had their tumors detected by ultrasound. Of these patients, 56% had their tumors detected on studies done to evaluate the thyroid for non-nodule related reasons and 54% had their tumors detected on studies done to evaluate structures other than the thyroid. Of the patient discovered by palpation, 84% were initially palpated by a physician and 16% were initially palpated by the patient.

Average follow up was 30 months. There was a recurrence in 5.2% of cases. There was no difference in recurrence rates in the imaging, palpation, and incidental group (3.6%, 7.4%, and 2.6% respectively, p-value 0.31). This was also true when excluding the incidental group (p-value 0.26) and after adjusting for confounding (p-value 0.28). There was also no difference noted between the obese and non-obese groups (4.5% and 5.5% respectively, p-value 0.260). Further, in our previous study we showed that imaging modality had no relationship to tumor stage or nodal status [13].

Among the patients in whom BMI was known, 181(67%) were in the Non-obese Group and 89 (33%) in the Obese Group. The comparison of these two groups is summarized in Table 2. There was no significant difference in the patient or tumor characteristics, or in the way in which the tumor was initially detected. Multinomial regression was used to adjust for confounding by age, sex, and tumor size, and there was no significant relationship between the Obese and Non-obese Groups in regards to detection modality (p-value 0.701, 0.628 for imaging and palpation groups respectively; reference group incidental tumors).

**Table 2:** Comparison of Obese patients to Non-obese patients.

	Non-obese	Obese	
	181 (67%)	89(33%)	P-value
Average Age	53(±14)	51(±12)	0.33
Female	130(72%)	60(67%)	0.46
Detection Method			
Palpation	81(45%)	40(45%)	
Imaging	72(40%)	38(43%)	
Incidental	28(16%)	11(12%)	0.77
Average Size (mm)	16 (±13)	18.3(±13)	0.26
Tumors <1cm	60(33%)	23(26%)	0.22

n = 270.

When a secondary analysis was performed with patients divided into 4 BMI categories, there were no significant differences in patient or tumor characteristics, or in initial tumor detection method (Table 3). There was a difference between groups in regards to the gender distribution. This was also true after performing a multinomial logistic regression with age, sex, and tumor size as confounders. We also performed a further analysis with additional BMI groups (BMI <25 kg/m^2^ and BMI 25–29.9 kg/m^2^) and similar results were found.

**Table 3:** Comparison of BMI groups.

	BMI	BMI	BMI	BMI	
	<30	30–34.9	35–39.9	>40	P-value
	181(67%)	51(19%)	20(7%)	18(7%)	
Average Age	53(±14)	53(±12)	47(±11)	50(±13)	0.26
Female	130(72%)	29(57%)	14(70%)	17(94%)	0.02
Detection method					
Palpation	81(45%)	26(51%)	8(40%)	6(33%)	
Imaging	72(40%)	19(37%)	8(40%)	11(61%)	
Incidental	28(16%)	6(12%)	4(20%)	1(6%)	0.56
Ave Size (mm)	16 (±13)	19(±13)	16(±10)	19 (±18)	0.55
Tumors <1cm	121(67%)	40(87%)	13(65%)	13(72%)	0.43

n = 270.

At the time of surgical consultation 53 (48%) of the 110 tumors initially found by imaging were actually palpable by the attending surgeon. These tumors were more likely to be palpable in Non-obese Group compared to Obese Group (57% vs. 32% respectively, p-value 0.025). The Non-obese and Obese Groups were otherwise similar in this subset of patients (Table 4). In the cancers detected by imaging, it is important to note that there was no significant difference in size or in the percentage of tumors <1cm when the Non-obese and Obese Groups were compared.

**Table 4:** Comparison of Obese patients to Non-obese patients among those detected by imaging.

	Non-obese	Obese	P-value
Average Age	54(±14)	55(±13)	0.61
Female	45(63%)	25(66%)	0.73
Palpable at consultation	41(57%)	12(32%)	0.03
Average Size (mm)	17(±13)	21(±14)	0.13
Tumor<1cm	19(26%)	6(16%)	0.21

n = 110.

## Discussion

This study demonstrates that the common sense hypothesis that obese patient are less likely to have thyroid tumors detected by palpation is not correct. Patients who were obese were just as likely to have their cancers detected by palpation as those who were not obese. This lack of correlation was observed when the patient population was divided into Obese versus Non-obese Groups and when the obese patients were further characterized by degree of obesity. There was also no difference seen in the size of the tumors or in the proportion of tumors < 10 mm when stratifying patients by BMI.

The incidences of WDTC and of obesity have been increasing in the United States [1–5, 14]. The increasing incidence of WDTC is probably due to over diagnosis [1, 7–12]. Almost 50% of WDTC is initially discovered on imaging studies [13, 20] and a correlation has been shown between frequency of ultrasonography and WDTC incidence [21]. Further, an association between obesity and the incidence of WDTC has been reported [15–17]. It has been suggested that the association of obesity and WDTC may be due to insulin like growth factors, adipokines, inflammation, and sex hormones [14]. However, it also seems reasonable to assume that palpation of thyroid nodules might be more difficult in obese patients. If this were true, one would expect that a greater percentage of WDTCs would be detected by imaging in obese patients compared to non-obese patients. With increasing numbers of imaging studies detecting more WDTCs, it would then appear that the rate of increase of WDTC in obese patients was higher than in non-obese patients. This study shows that this hypothesis is not correct.

Interestingly, when looking at the subset of patients initially detected by imaging, obesity did result in tumors being less likely to be palpable at the time of initial surgical consultation. This partially confirms the initial hypothesis that it is more difficult to palpate a nodule in an obese patient. In spite of this, the percentage of cancers initially detected by palpation was the same in obese and non-obese patients and was independent of the degree of obesity.

Our previous study demonstrated that the initial detection of WDTCs by imaging is not limited to small cancers. The percentage of large cancers detected by imaging was almost as great as the percentage of small cancers [13]. This was an unexpected observation. We were concerned that the large proportion of tumors found by imaging, particularly among larger tumors, was related to a higher BMI in these patients. This does not appear to be true.

Our results suggest that any relationship between increasing BMI and WDTC rates is not due to a difficulty in palpating small tumors in patients with a higher BMI or to the way in which the tumors are initially detected. Other factors that may explain the apparent association between obesity and WDTC could include differences in access to medical care in obese populations compared to non-obese populations, differences in frequency of medical visits due to coexisting medical conditions, or a true causal link between obesity and thyroid cancer. Although a recent pooled analysis and separate large meta-analysis both found an association between body mass index and thyroid cancer risk, a true causal relationship has not yet been proven [15, 22]. In a study of obesity-related genetic polymorphisms, there was no association with thyroid cancer risk [23].

This study has some limitations. This is an observational study and there may be unmeasured confounding variables that we did not take into account. Patients were referred by a variety of physicians and centers. We do not have data on the practice patterns of the referring physicians, medical co-morbidities, or frequency of medical visits. Different referring physicians and centers may have differed on frequency and sensitivity of ultrasound and physical thyroid examination. Further, we do not know if the referring physician was an endocrinologist or primary care physician. When we included age, sex, and gender, however, we did not see any change in the relationships we observed. We did not measure cervical circumference, but used BMI as a surrogate marker. However, these two variables have been found to be highly correlated [24]. We had to exclude a large number of patients because of missing BMI data. The excluded patients were younger. This could have introduced bias into the study and our findings may not be generalizable to younger patients. Furthermore, the excluded patients may have had lower BMIs, as operating room staff may be less likely to record height and weight for healthy appearing patients. This could dampen any effect that BMI had on detection modality. In order to assess the magnitude of this bias, we performed two sensitivity analyses. In one we assumed that all patients with missing BMI data were obese and in the other we assumed that they were not obese. In both cases the relationship between obesity and imaging modality was not statistically significant (p-values 0.685 and 0.561, respectively). Our study may have also suffered from recall bias, as patients were asked at their initial surgical consultation to recall how their tumors were initially detected. Our sample size was also relatively small, and we may have detected a statistical difference with a larger study population. However, the observed percentages of tumors found by palpation in the Obese and Non-obese groups, 44.9% and 44.7% are so similar that this is unlikely. Lastly, this study only included patient who underwent surgery for their lesions.

In conclusion, we show that BMI does not play a role in initial detection modality of WDTCs. This suggests that the prevalence of WDTC detected by imaging is not an artifact caused by an increasingly obese population and that any association of WDTC and obesity is not related to the way in which these tumors are detected. Future studies examining the relationship of BMI and WDTC rates should take this into account.

## Supporting Information

S1 File This file contains the data used for statistical analysis. (XLSX)
